# Clinical profile of sickle cell disease in children treated at “Cliniques Universitaires de Bukavu” and “Clinique Ami des Enfants”, Bukavu, Democratic Republic of the Congo

**DOI:** 10.11604/pamj.2022.41.97.29629

**Published:** 2022-02-03

**Authors:** Viviane Feza Bianga, Mwanza Nangunia, Fernand Manga Oponjo, John Mambo Itongwa, Judicaël Iragi Mushubusha, Moise Mbaluku Colombe, Carmel Mbalo Walemba, Okitosho Wembonyama

**Affiliations:** 1 Service of Pediatrics, Cliniques Universitaires de Bukavu, Official University of Bukavu, Bukavu, Democratic Republic of the Congo,; 2 Department of Pediatrics, Faculty of Medicine, Official University of Bukavu, Bukavu, Democratic Republic of the Congo,; 3 Clinique Ami des Enfants, Bukavu, Democratic Republic of Congo,; 4 Club Univers Médical of Medicine Faculty, Official University of Bukavu, Bukavu, the Democratic Republic of the Congo,; 5 Department of Pediatrics, Faculty of Medicine, University of Lubumbashi, Lubumbashi, the Democratic Republic of the Congo

**Keywords:** Sickle cell disease, children, Bukavu, Democratic Republic of the Congo

## Abstract

**Introduction:**

sickle cell disease is the most common inherited hemoglobin disease globally and remains a significant concern worldwide and in Africa. This study aimed to determine the clinical profile of children with sickle cell disease followed up in two hospitals in Bukavu.

**Methods:**

we conducted a cross-sectional multicenter retrospective study. Medical folders of children with sickle cell disease followed up at the Cliniques Universitaires de Bukavu and Clinique Ami des Enfants, collected from January 2018 to December 2019, have been used.

**Results:**

in 55 sickle cell children, 31 cases (54.4%) were male against 24 (43.6%) females. The average age was 101.09 months (between 12 and 222 months). Diagnosis has been established before two years, with an average age of 14.27 months. The main circumstance of the discovery of the disease is anemia. Electrophoresis was the primary test of diagnosis in 81.8% of cases. Transfusion was done an average of 5.98 times (0 and 10 times) during different treatment period. Anaemia and infectious phenomena were encountered respectively in 96.4% and 96.4%. However, acute chest syndrome was only found in 9.1%.

**Conclusion:**

sickle cell disease has been diagnosed precociously before two years and anemia was the main circumstance of discovery. A better knowledge of caregivers about the various clinical aspects and an early screening could improve the quality of life of these children.

## Introduction

Sickle cell disease is the most common hereditary disease globally and constitutes a real public health problem on a continental scale in Africa [1]. There are two primary forms of the sickle cell trait, including the heterozygous form AS and the homozygous form S.S. However, heterozygous composite forms also exist and can be associated with other blood diseases [2]. Clinically, there are three types of signs and symptoms related to sickle cell disease, particularly chronic hemolytic anemia, vaso-occlusive crisis, and extreme susceptibility to infections [3].

The World Health Organization estimates nearly 50 million people with a sickle cell trait globally with a frequency reaching 38 million in sub-Saharan Africa; Africa has 500,000 children with sickle cell disease, and 70% die before the fifth year of their birthday due to lack of adequate care. The frequency of the sickle cell trait determines the prevalence of the disease at birth. This trait varies depending on whether you are in an area, so areas endemic to Malaria have a high frequency of sickle cell disease [4]. In Africa, the frequency of sickle cell trait can reach 40% and varies from west to east, while increasing, for example, in Nigeria, the most populous country in Africa, where 24% of the population are a carrier of the sickle cell trait and in Senegal where 15% of the population is a carrier, against 40% in Central Africa [5,6].

In the Democratic Republic of the Congo, around 40,000 births of children with sickle cell disease are recorded each year, and 2% of these newborns are homozygous. This disease remains little known to the population, which explains the high mortality in our country with limited resources. Furthermore, in 2008, a study carried out in 5 provinces (Kinshasa, Katanga, Orientale Province, Bas-Congo, and the Kasaï) reported a frequency of more than 17% in children screened from birth [5,7]. Given the scope of sickle cell disease in DRC and the absence of data in South Kivu, a region in the east, it is opportune to conduct this study to determine the clinical profile of children affected with sickle cell disease. These data could raise awareness and be a basis for advocacy to intensify the fight against sickle cell disease in South Kivu.

## Methods

**Study design:**this study is a cross-sectional multicenter and retrospective study.

**Setting:** this study was carried at the “Cliniques Universitaires de Bukavu” (hospital affiliated to the Official University of Bukavu) and “Clinique Ami des Enfants,” both located in Bukavu City, South-Kivu Province, in Democratic Republic of Congo. Bukavu is a city situated on the shores of Lake Kivu, the capital of the province of South Kivu, located in the east of the Democratic Republic of the Congo, i.e. 2° 30' 55'' south and 28° 50' 42'' east, with an area of 60 km^2^. The city is inhabited by an estimated population of 2,000,000 in 2019.

**Participants:** we extracted data from medical charts of patients followed up at those hospitals from January 2018 to December 2019 and included children with sickle cell disease in this study. Were excluded from this study, all children not followed up for sickle cell disease, and those whose follow-up started in 2019.

### Variables

**The following variables studied were:** sociodemographic profiles: age, gender, level of study of children; clinical features: history (age of discovery of the disease, age of the first attack, circumstances of the discovery of the condition, hemoglobin value at the time of the consultation in g/dl, number of transfusions, diagnostic assessment) and the various significant syndromes: acute chest syndrome, infectious episodes, anemia, vaso-occlusive crisis, etc.

**Study size:** the sample was exhaustive for convenience and included all the sickle cell children followed up in those two hospitals for the period of this study. Our study had 55 children followed up at the Cliniques Universitaires de Bukavu and Clinique Ami des Enfants.

**Quantitative variable:** for the quantitative variables, we determined the mean, the standard deviation and studied the correlation.

**Statistical methods:** data were analyzed by using SPSS version 20 and Stata version 8 S.E. software; the frequency comparison was performed by the “t-student” test at the statistical significance level p < 0.05 and p < 0.001 (95% confidence interval); we calculated the correlations by using the Stata software.

**Ethical consideration:** the study was approved by the ethics committee of the Official University of Bukavu, according to international guidelines for research ethics. Data analysis and processing were anonymous and confidential.

## Results

### Descriptive data

**Sociodemographic status:** Table 1 represents the sociodemographic status of 55 children with sickle cell treated in those two hospitals. This table indicated that 31 (54.4%) among 55 patients treated in those hospitals are males against 24 patients (43.6%) who are females. Referring to age, our results show that the mean age of the children in months is 101.09, with the extremes ranging between 12 months and 222 months. So, as represented in Table 1, 70.9% of children are over than 60 months old, and only 7.3% have age under or equal to 24 months. 54.5% of children have a primary education level, and 34.5% have no education level (Table 1).

**Table 1:** sociodemographic status of patients

Variables	Effective	Percentage	Mean (SD)
**Sex**			
Male	31	56.4	101.09 (54.61)
Female	24	43.6	101.09 (54.61)
**Age (in month)**			
≤ 24	4	7.3	101.09 (54.61)
24 and 60	12	21.8	101.09 (54.61)
> 60	39	70.9	101.09 (54.61)
**Education level of children**			
Primary	30	54.5	101.09 (54.61)
Secondary	6	11	101.09 (54.61)
None	19	34.5	101.09 (54.61)

### Main results

**Paraclinical and clinical profiles:** in Table 2, all paraclinical and clinical parameters are represented. Results in the above table demonstrate that mean age of 14.27 months of diagnosis of the disease (3 months and 66 months), attacks begin early, 85.5% were less than or equal to 24 months; the correlation of the mean age at onset of the first attack and the child's age at the first attack was statistically significant (p < 0.001). The primary diagnostic circumstance is anemia, which was found in 25 children representing 45.5%, followed by vaso-occlusive crises in 10 children or 20%, however, other circumstances such as jaundice, respiratory difficulty and splenomegaly, were only found in 2 children, i.e. 3.6%. As diagnostic testing methods, hemoglobin electrophoresis was the main and found to be used in 81.8%. However, the Emmel test has been used only in 18.2% of cases. The mean hemoglobin level was 6.425 g/dl (2.4 g/dl and 10 g/dl, deviation standard 1.59), and 80.0% (44 children) of patients had a hemoglobin level between 5 g/dl and 10 g/dl; the correlation of hemoglobin value and the child's age at the first attack was also statistically significant (p < 0.001). The transfusion was performed on average 5.98 times (0 and 10 times, deviation standard 4.9) during different treatment period a year, 23.6% or 13 children had never been transfused against 27.3% or 15 children who have already been transfused between 5 times and ten times and 25.5% or 15 children were transfused more than ten times.

**Table 2:** paraclinical and clinical parameters of children with sickle cell disease

Variables (min and max)	Effective	Percentage	Mean (SD) and p-value
**Age of diagnostic (in month)**			**14.27 (13.72) 0.0001 (actual age in month)**
≤ 24	47	85.5	
> 24	8	14.5	
**Circumstances of diagnostic**			
Anemia	25	45.5	
Vaso-occlusive crisis	11	20.0	
Infection	7	12.7	
Associated circumstances	10	18.2	
Other: icterus, breathing distress, splenomegaly...	2	3.6	
**Diagnostic testing methods**			
Emmel test	10	18.2	
Electrophoresis	45	81.8	
**Hemoglobin value at diagnostic (2.4, 10) (g/dl)**			
< 5	11	20.0	6.425 (1.59)
5 to 10	44	80.0	
**History of transfusion (0, 15) (number of times a year)**			**5.98 (4.90)**
None	13	23.6	
< 5	13	23.6	
5 to 10	15	27.3	
> 10	14	25.5	

**Major and mixed syndromes:** results from Table 3 show that the most syndromes found in patients are anemic and infectious, representing 96.4% of all cases, i.e. 53 times during different treatment periods, while the acute chest syndrome represents only 9.1% or five times. Mixed syndrome SM2, a combination of three syndromes (anemia, infection, and acute thoracic syndrome), was found in 41.8% of cases.

**Table 3:** major and mixed syndrome

Variables	Effective	Percentage	p-value
**Major syndromes**			
Anemic	53	96.4	0.81 (sex)
Infectious	53	96.4	
Acute chest	5	9.1	
Vaso-occlusive crisis	30	54.5	
**Mixted syndromes**			
SM1	23	41.8	
SM2	27	49.1	
SM3	5	9.1	

**Other analysis:** we determine the correlation between the mean age and the first attack; event the correlation between hemoglobin and the age of children when the diagnosis was made.

## Discussion

Sickle cell disease is a group of conditions, which are inherited, and affect the blood and various organs in the body. It remains a global health problem in almost all countries worldwide, especially in developing countries affecting children. Africa and the Democratic Republic of Congo are not spared. Given the scope of sickle cell disease in DRC and the absence of data in South Kivu, a region in the east, it is opportune to conduct this study to determine the clinical profile of children affected with sickle cell disease.

### Keys results

**Sociodemographic characteristics of children:** male children are more concerned and represent more than half, i.e. 56.4%, the sex ratio of 1.2 M/F. Similar results were found in two studies on the clinical and hematological profile of children with sickle cell disease with sex ratio of 1.3 M/F and 1.4 M/F respectively in Ziguinchor and Dakar, Senegal [8,9]. An epidemiological and clinical study in children under 5 years of age conducted in Lubumbashi showed a predominance of females with a sex ratio of 1.1 F/M [10]. In Burkina-Faso, two studies respectively found a sex ratio of 0.92 M/F and a predominance of females [11,12]. In two other studies conducted respectively at the University Hospital of Grenoble in France and in a pediatric setting in Gabon, no gender predominance was observed [13,14]. It is crucial to notice that the transmission of sickle cell disease, according to whether encountered in the literature is not linked to sex but earlier in the demographic data of each country.

Referring to our results, the mean age of the children is 101.09 months (8 years and 4 months), with extremes of 12 months (1 year) and 222 months (18.5 years). Our observation is different from that found in a study on the clinical and epidemiological profile of SS homozygous sickle cell disease in the intercritical phase in children in Ziguinchor where the mean age was 8 years with extremes of 11 months and 21 years [9]. In this present study, children have a primary level for a half of all cases (55.5%). Our results are not similar to those found in a study conducted in three hospitals in Yaoundé, Cameroon on the epidemiological, clinical and therapeutic aspects associated with vaso-occlusive attacks in SS children where 47.1% of the children had a primary school education [15]. In another study in Dakar, Senegal, pupils and students represented 44.96% of patients [16]. This difference in level of education could be associated to a certain socio-economic level and to the repetition of crises that may justify irregular schooling due to absenteeism.

**Paraclinical and clinical features of children:** our study found that the diagnosis was established early, especially before 24 months in 85.5% of cases with a mean age of 14.27 months (extreme from 3 to 66 months). In children whose disease was diagnosed early (before 24 months), the mean age at their first attack was 10 months (95% CI 7.87-12.3) compared to 34 months. This difference is statistically significant (p < 0.0001). Our results are similar to those found in a study conducted in Lubumbashi where the mean age of the first attack was 10.1 months with a standard deviation of 10.0 (extremes of 3 and 54 months) [10]. While a study conducted at the General Referral Hospital in Kindu found that the diagnosis was made late with an average age of 9 ± 4 years [17]. In addition, the study conducted in Ziguinchor, Senegal, found that more than 50% of patients with sickle cell disease were diagnosed late, i.e. in 58.3% of cases with a mean age of 35.5 months (extreme from 7 to 192 months) [8]. Concomitantly, in two studies conducted respectively in Senegal and Abidjan, it was observed that the majority of children had their first attacks after the age of 2 years, and in their series, the majority of children (63.74%) had their attacks between 6 and 11 years of age [8,18]. This diagnosis difference depends on whether a nation has a birth control service for congenital disease as in the United States and United Kingdom [18].

According to our results, anemia is the main circumstance of discovery in 47.27% of cases. This result is similar to those found in a study on the blood count of Congolese sickle cell children where anemia and hand-foot syndrome were the circumstances of discovery [7]. In a study conducted in Ziguinchor, Senegal, the hand-foot syndrome was the reason for the discovery of the disease in 30.4% of sickle cell patients [8]. However, at the Laquintinie Hospital in Douala, Cameroon, vaso-occlusive crises were the reason for the discovery of sickle cell disease in 51.3% of patients, followed by anemia in 17.8% [19]. In addition, a study conducted in Burkina Faso on major sickle cell syndromes and associated infections in children, associated the discovery of the disease with infectious episodes in sickle cell patients [11]. Bone and joint pain led to the discovery in a study conducted at the University Hospital of Abidjan [18]. In an epidemioclinic study at Lubumbashi among sickle cell children aged 6-59 months, more than half (95.2%) had hand-foot syndrome or dactylitis as their first complaint [10]. A study conducted in Dakar, Senegal, concluded that osteoarticular pain (34.8%) followed by hand-foot syndrome (14.5%) was the first complaint of sickle cell disease [9]. In addition, a study conducted in the Pediatric Department of the Gabriel Touré University Hospital in Bamako, Mali, found osteoarticular pain to be a warning sign in the discovery of the disease [20].

Our results found electrophoresis as the primary diagnostic testing methods in the majority of cases (78.18%). This result correspond to those found in a study on the epidemiological and clinical profile of children and adolescents with major sickle cell syndromes admitted in emergency situations to pediatric consultations in Dakar. Furthermore, it is different from those found in a study on the causes of hospitalization of children with sickle cell disease at the Centre Hospitalo-Universitaire in Brazzaville where the Emmel test (*in vitro*sickling test) used to the diagnosis in 46.03% of cases before confirming it by electrophoresis of hemoglobin [21]. In our series, the mean number of transfusions is 5.98 (from the first time of the diagnosis to date) with the extremes of 0 to 15 numbers of transfusions. Our results are different from those found by many others authors: in Senegal, half of the patients had already been transfused, with an average transfusion of 0.5 and extremes ranging from 0 to 4 times the number of transfusions [8]; and at the Centre Hospitalo-Universitaire of Abidjan, 32% of the patients were transfused once and the others more than twice [18].

However, in a study conducted in Lubumbashi, the average number of transfusions was 2.1 with a standard deviation of 2.30 and also, almost 4/5 of the patients were transfused at least once with extremes of 0 to 9 times the number of transfusions [10]. In a study conducted at the Central Hospital of Yaoundé in Cameroon, 86.1% of patients had been transfused at least once before, and 13% had been transfused more than 10 times [22]. Our study showed that in 50% of patients, hemoglobin at the time of consultation was 7g/dl with extremes ranging from 3-10 g/dl. Our results are similar to those found in several studies, 7.0 g/dl and 7.4 g/dl respectively in Lubumbashi and Kinshasa [23].

However, there is a slight increase in the hemoglobin level in female children (Hb at 7.3 g/dl) compared to male children (Hb at 6.8 g/dl), the difference not being statistically significant (p > 0.05) [23]. This is different from what was found in a study conducted in Bamako, Mali, where the hemoglobin level ranged from 4-6 g/dl with a mean of 4 g/dl and a standard deviation of 1.5 [24]. In a study conducted at the University Hospital of Abidjan, it was found that the majority of patients (70%) were transfused with Hb levels of 4 and 5 g/dl; no sickle cell patient was transfused with 7 g/dl Hb [18]. In Lubumbashi, in a study of children with sickle cell disease, the hemoglobin level varied between 5.6 and 10.5 g/dl with a mean and standard deviation of 8.3 ± 1.4 g/dl [10]. In Dakar, Senegal, a similar study showed that the hemoglobin level on admission averaged 7.9 g/dl (SD 1.6) with extremes of 3.3 and 12 g/dl [9]. Similar results were found in two studies of sickle cell disease with a mean hemoglobin level of 7.4 g/dl, SD 1.4 for homozygotes and a mean baseline hemoglobin level of 7.9 ± 1.01 g/dl respectively in the pediatric department of Lomé in Togo and Dakar in Senegal [25,26]. The anemia is explained by chronic hemolysis in children with sickle cell disease.

Major and mixed syndrome: our series indicated that infectious episodes and anemia are the major syndromes most frequently found with a percentage of 96.4% for each major syndrome. Similar fundings were obtained, in a study conducted in Ziguinchor, Senegal, where 95.6% of patients had chronic anemia [8]. In another study conducted in Congo Brazzaville, painful attacks with bone and/or joint localization were the most common [27]. In addition, pure abdominal pain attacks were the third most common, accounting for nearly 16% of cases. Nevertheless, a study conducted in three hospitals in Yaoundé, Cameroon, found abdominal attacks in patients from 1 to 15 years of age, hand-foot syndrome was significantly present in children under 5 years of age, and long bone and spine involvement in children over 5 years of age [15]. In our study, there is no statistically proven difference in sex according to major syndrome (p < 0.81). This difference is not also found in other studies: at the University Hospital of Abidjan where osteoarticular pain was the most frequent followed by anemia, but without any sex difference [18]. Furthermore, in a study conducted in India, acute infectious syndrome is frequent, being the most frequent pathological event followed by severe anemia syndrome and other syndromes [3], no statistically proven difference was found in this distribution. Likewise, there is no statistically proven difference in associated major syndromes according to the current age of the children, the mean age of those with 2 syndromes being 92.8 ± 57.2 (69.7 - 115.9) and that of those who had 3 and more syndrome is 110.9 ± 51.6 (91.3 - 130). Compared to the results found from Brazzaville University Hospital Center [21], infections represented 36.6% of all admissions; they were as frequent before 5 years of age as after that age, with p > 0.05.

In a study conducted in Dakar, Senegal, on the epidemiological and clinical profile of children and adolescents with major sickle cell disease admitted to emergency pediatric consultations, peaks in the frequency of vaso-occlusive, infectious and anemic crises were generally observed in children aged 5 to 10 years [25].

**Limitations:** we recognize that this study had limitations. We had a low sample. If it was only a prospective study, it could give an excellent evolution of children with sickle cell disease.

## Conclusion

This study shows a male predominance, and the diagnosis was established precociously before two years; anemia was the main circumstance of discovering the disease. The transfusion has been done many times during different treatment periods. Major syndrome, infection, and acute chest syndrome are most frequently found as reported in this multicenter, cross-sectional study. A better knowledge of caregivers about the various clinical aspects and an early screening could improve the quality of life of these children.

### What is known about this topic

- Sickle cell disease remains a public health problem, particularly in the Democratic Republic of Congo where prevalence is high;
- In the province of South Kivu, little data exists concerning sickle cell disease.

### What this study adds

- At Cliniques Universitaires de Bukavu and Clinique Amis des Enfants, children with sickle cell disease are known and followed;
- For these children, the diagnosis is made early, before 2 years old, and anemia remains the leading circumstance of discovering the disease.
